# Comparison of immunotherapy combined with stereotactic radiotherapy and targeted therapy for patients with brain metastases: A systemic review and meta-analysis

**DOI:** 10.1515/biol-2022-0559

**Published:** 2023-03-01

**Authors:** Zhou Su, Li Zhang, Shaolong Xue, Youke Wang, Ruining Ding

**Affiliations:** Department of Oncology, Sichuan Mianyang 404 Hospital, Mianyang, Sichuan 621000, China; Department of Oncology, West China School of Medicine, SCU, Chengdu, China; Department of Oncology, Chengdu University of Traditional Chinese Medicine Affiliated Hospital, Chengdu, Sichuan, P.R. China; Department of Oncology, Institute of Drug Clinical Trial/GCP Center, Affiliated Hospital of Southwest Medical University, Luzhou, Sichuan 646000, China

**Keywords:** brain metastases, immunotherapy, radiotherapy, meta-analysis, overall survival

## Abstract

Advances in brain imaging have led to a higher incidence of brain metastases (BM) being diagnosed. Stereotactic radiotherapy (SRS), systemic immunotherapy, and targeted drug therapy are commonly used for treating BM. In this study, we summarized the differences in overall survival (OS) between several treatments alone and in combination. We carried out a systematic literature search on Pubmed, EMBASE, and Cochrane Library. Differences in OS associated with Immune checkpoint inhibitors (ICI) alone versus targeted therapy alone and SRS + ICI or ICI alone were evaluated. This analysis was conducted on 11 studies involving 4,154 patients. The comprehensive results of fixed effect model showed that the OS of SRS + ICI group was longer than that of the ICI group (hazard ratio, 1.72; 95% CI: 1.41–2.11; *P* = 0.22; *I* ^2^ = 30%). The combined fixed-effect model showed that the OS time of ICI was longer than that of targeted therapy (hazard ratio, 2.09; 95% CI: 1.37–3.20; *P* = 0.21; *I* ^2^ = 35%). The study had a low risk of bias. In conclusion, our analysis confirmed that immunotherapy alone showed a higher OS benefit in BM patients than targeted therapy alone. The total survival time of patients with SRS combined with ICI was higher than that of patients with single ICI.

## 1 Introduction

About 20% of cancer patients will have brain metastasis (BM) during the clinical process associated with the disease, especially melanoma, lung cancer, and breast cancer [1]. This number is expected to increase due to improvements in the early detection of brain metastases (BM) and the efficacy of systemic treatment. However, the prognosis of BM has not been optimistic. The median survival time without treatment was estimated to be 1 month. Radiotherapy is one of the most commonly used methods of treatment for BM, in part because few systemic drugs can penetrate the blood–brain barrier [2]. When brain radiotherapy is used, the survival rate increases up to 3–12 months [3]. The conventional method of treatment for patients with isolated BM is resection plus whole-brain radiotherapy (WBRT) [4]. Previous studies have shown that adjuvant WBRT after resection can improve intracranial tumor control and reduce neurological mortality compared with resection alone [5,6].

Immune checkpoint inhibitors (ICI) have achieved excellent results in the treatment of different cancers, including melanoma and non-small cell lung cancer (NSCLC). These ICIs target cytotoxic T lymphocyte-associated protein 4 (CTLA-4) and programmed death (ligand) 1 (PD-(L)1) molecules on T cells, leading to the prolonged activation of T cell response and subsequent stimulation of antitumor activity [7]. Margolin et al. showed a 24% tumor control rate in melanoma BMs treated with ICI monotherapy in patients with BM [8]. The use of ICI in combination with stereotactic radiosurgery (SRS) in BM patients showed higher intracranial response rates and better survival than ICI alone [9,10]. In addition to ICI, the use of molecular targeted therapy also showed BM activity [11]. Targeted therapies, such as common HER2/ERBB2 inhibitors, included trastuzumab and pertuzumab. The advantage of trastuzumab was that it could improve the outcomes of breast cancer with BM, as had been widely demonstrated. Trastuzumab was effective and well tolerated in patients with metastatic breast cancer and BM, as evaluated in the KAMILLA Clinical trial [12]. Predicting the existence of gene changes, such as epidermal growth factor receptor mutation or BRAF V600E mutation, had been considered to be a necessary prerequisite for targeted treatment response [13].

In order for clinicians to make better treatment choices, it is necessary to summarize the effects and outcomes of these treatments. To clarify this issue, we conducted a systematic review and meta-analysis of the currently available literature on the total survival (OS) of BM patients using targeted therapy, radiotherapy, and immunotherapy.

## 2 Methods

### 2.1 Literature search strategy

This meta-analysis was performed following the Preferred Reporting Project for Systematic Reviews and Meta-Analyses (PRISMA) statement [14]. The review protocol we used had not been published and was not pre-registered. All data for the current study were available from the corresponding author upon reasonable request. A systematic literature search was conducted on PubMed, Embase, and Cochrane Library until February 2022. References included in the study were examined to identify other relevant publications. Study screening and data extraction were performed by two independent reviewers (ZS and LZ) according to the PRISMA checklist. In case of discrepancies, a third examiner (RD) was consulted. The terms “brain neoplasms,” “Medical subject heading” (“MeSH”), and “metastatic brain” were combined with the MeSH terms “Radiotherapy,” “Targeted therapy,” and “Immunotherapy.” We also searched relevant literature reviews to find any other eligible articles.

### 2.2 Selection criteria

Inclusion criteria were as follows: (1) For any study design, at least 10 participants had to be included in each group; (2) patients with BM were used as the study population, and radiotherapy, targeted therapy, or immunotherapy was used as at least one of the treatment methods; (3) the prognosis of patients with BM after relevant treatment was reported. The main outcome of this study was median overall survival (OS); (4) survival data or the Kaplan–Meier curve was provided in the study. The exclusion criteria were (1) studies conducted on animals; (2) in the absence of substantial brain metastasis, only studies on light meningeal diseases were reported; (3) there was no full-text research; and (4) non-English publications.

### 2.3 Data extraction

The following information was extracted: study characteristics including study design and sample size, patient gender, age, and primary tumor site; treatment characteristics including previous craniotomy and/or radiotherapy, type of immunotherapy and targeted therapy; and clinical outcome. When no additional unpublished data was presented, the authors should be contacted.

### 2.4 Quality assessment

The quality of the cohort studies was assessed using the Cochrane Collaboration’s tool [15]. Two authors (ZS and LZ) independently performed the evaluation. Differences in the evaluation were resolved through discussion.

### 2.5 Statistical methods and publication bias

When *I* ^2^ > 50%, *P* < 0.1, the random effects model was chosen; when *I* ^2^ < 50%, *P* > 0.1, the fixed effects model was chosen [16]. By applying the “weighted median of the median” method (McGrath et al., 2019), the specific median OS of the study was combined into the aggregate value and the corresponding 95% confidence interval [17]. The Chi^2^ value was calculated to perform the statistical heterogeneity test, and *I* ^2^ was used for quantitative statistics (Higgins et al., 2003). *I* ^2^ value > 50% was considered to indicate high heterogeneity [18]. The Cochrane Q test was used to assess the *P* values for heterogeneity (significant *P* values <0.1). Sensitivity analyses were also performed, including only studies that adjusted for confounders. To assess potential publication bias, funnel plots and Egger linear regression tests (Chaimani et al., 2013) were used for results [19]. All statistical analyses were completed using R version 4.2.1 (R Core team, Vienna, Austria). The “metagen” package was used for data analysis in this meta-analysis. *P* values of less than 5% were considered significant unless otherwise stated. During the process of data selection and quality evaluation, any discrepancies between reviewers were resolved through discussion.

## 3 Results

### 3.1 Study selection and study characteristics

**Figure 1:**
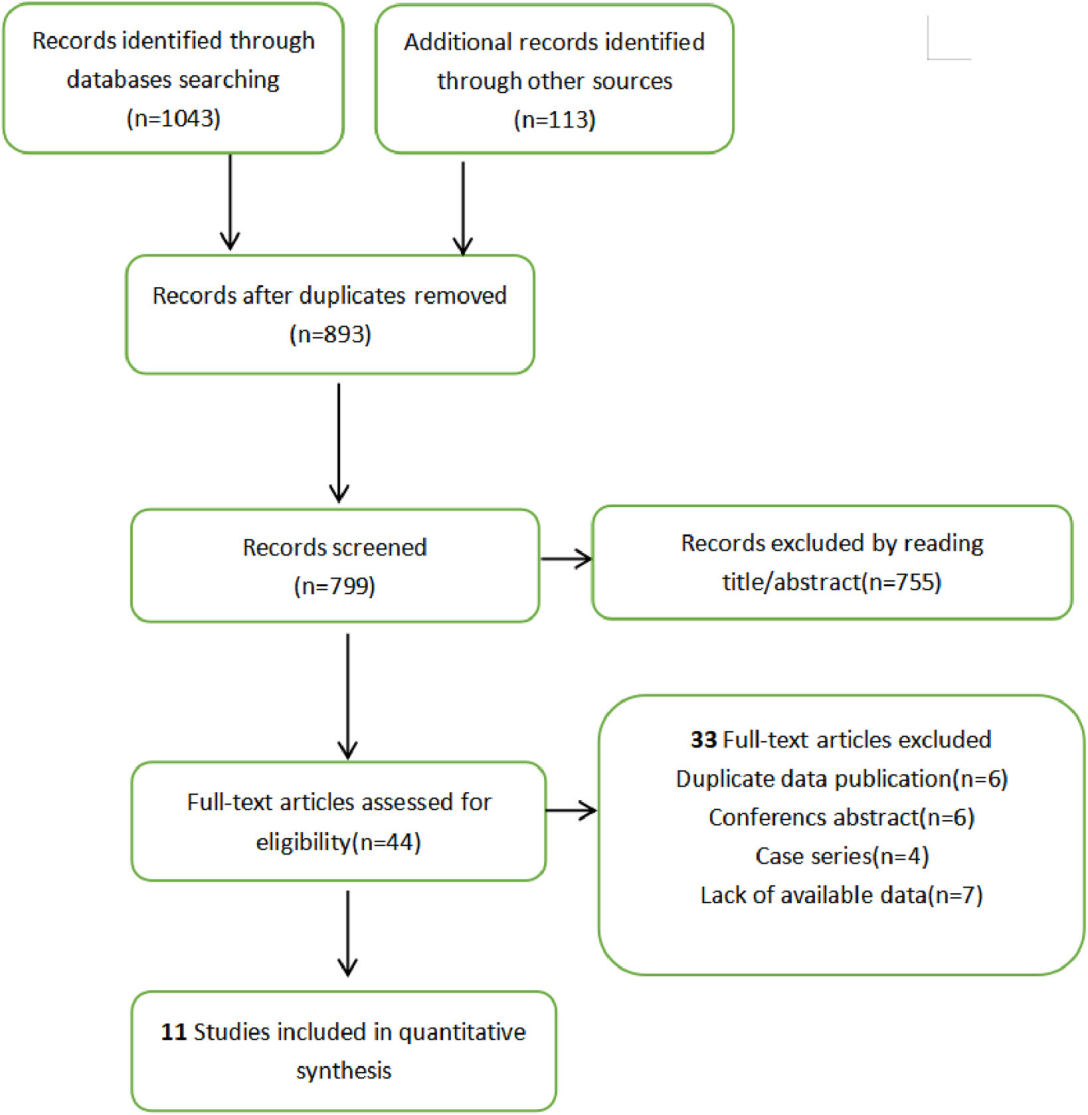
Flow chart.

**Figure 2:**
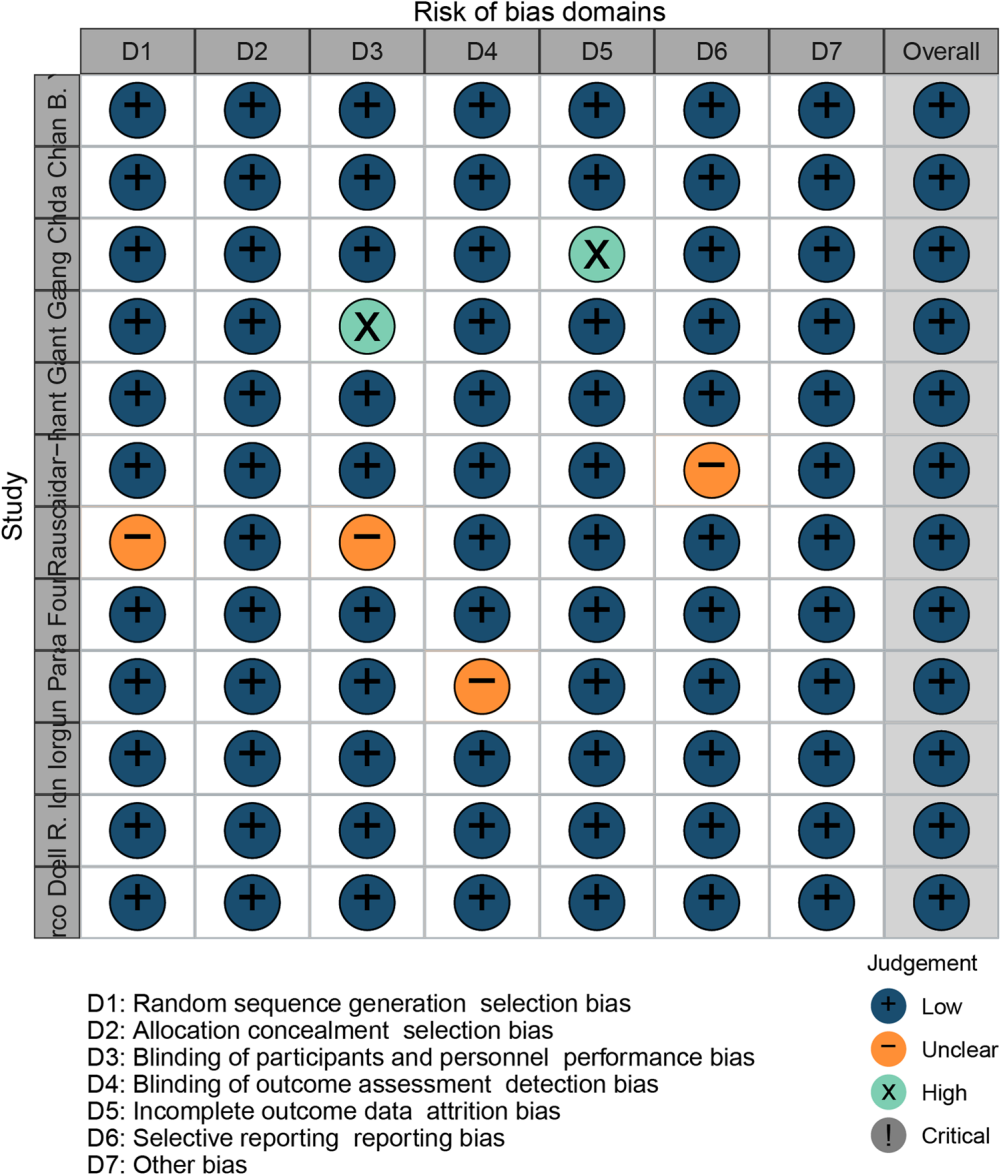
Risk of bias map.

The search strategy generated 893 articles after deleting duplicates. After screening the title and abstract, 44 full texts were taken forward for detailed screening. Finally, 11 studies were included after reading the full text. Figure 1 was the screening flow chart. Five cohort studies [20–24] explored the difference in total survival time between SRS + ICI and ICI alone. Three studies [25–27] explored the OS differences between ICI and targeted therapy. In the included studies, the ICIs used are Pembrolizumab, Ipilumumab, and Nivolumab. The targeted therapies used were BRAF inhibitors and MEK inhibitors. Subgroup analysis was conducted in three studies [26,28,29] to explore the total survival time of ICI and non-ICI. The total number of people involved was 4,154. The quality evaluation table is shown in Figure 2. The overall risk of publication bias in included articles was low. The specific characteristics of each document are presented in Table 1 (Figures 1 and 2; Table 1).

**Table 1:** Characteristics of included studies

First author, Publication year	Tumor type	Type of versus	Median age	Number	Median OS (95% CI)	*P*	HR	lci	uci
Mehran B. Yusuf, 2017 [20]	Melanoma	SRS + ICI	63.8 (37.1–81.9)	18	7.1 (1–51.8)	0.212	0.96	0.44	2.8
		SRS	63.6 (35.2–82.5)	33	7.4 (0.9–26.4)		1		
Linda Chen, 2017 [21]	Melanoma, NSCLC and Renal Cell Carcinoma	SRS + ICI (concurrent + ICI)	NR	28	24.7 (4–63)	0.006	0.37	0.2	0.7
		SRS + ICI (non-concurrent + ICI)	NR	51	14.5 (2–40)				
		SRS	NR	181	12.9 (2–55)	0.002	1		
Ee Siang Choong, 2017 [22]	Melanoma	SRS + ICI	NR	339	NR	NR	0.51	0.25	1.05
		SRS	NR		NR		1		
Prashant Gabani1, 2018 [23]	Melanoma	WBRT + ICI	62.0 (18–90)	103	8.5 (6.5–10.5)	＜0.001	0.74	0.61	0.88
		WBRT		638	4.4 (3.9–4.9)		1		
Prashant Gabani2, 2018 [23]	Melanoma	SRS + ICI	62.0 (18–90)	89	17.0 (10.7–23.2)	＜0.001	0.65	0.51	0.84
		SRS		274	11.9 (9.8–14.0)		1		
Orit Kaidar-Person, 2017 [24]	Melanoma	SRS + ICI	NR	29	15 (10–15.8)	0.0013	0.38	0.2	0.72
		SRS	NR	29	6 (3.8–8.4)		1		
Ricarda Rauschenberg, 2019 [25]	Melanoma	ICI	NR	138	14.8 (9.9–19.7)	0.03	3.2	1.7	6.3
		Target (BRAF inhibitor)	NR	67	9.8 (7.8–11.7)		1		
Andrea Fourscore, 2016 [26]	Melanoma	ICI	NR	10	14 (5.4–22.6)	0.001	1.82	0.79	4.19 (vs Tagert)
		Target (BRAF,MEK inhibitor)	NR	24	7 (0–17.9)		0.65	0.29	1.45 (vs non-IM)
		MEK inhibitor							
		Non-ICI (chemotherapy)	NR	23	9 (4.3–13.7)		1		
Sagun Parakhv, 2018 [27]	Melanoma	ICI	NR	21	NR	NR	1.34	0.63	2.83
		Target (BRAF inhibitor)	NR	15	NR		1		
J. Bryan Iorgulescu, 2018 [28]	Melanoma	ICI	NR	286	56.4(25–not reached)	＜0.001	0.12	0.03	0.49
		Non-ICI	NR	1,441	7.7(6.7–8.7)		1		
Russell R. lonseR, 2011 [29]	Melanoma	ICI	NR	15	19.1 (4.2–64.7)	0.06	0.58	0.24	1.41
		Non-ICI	NR	26	12.2 (2.3–95.2)		1		
Marco Donia, 2017 [30]	Melanoma	ICI	NR	276	NR	NR	0.36	0.25	0.52
		Non-ICI	NR		NR		1		

ICI, immunocheckpoint inhibitor; non-ICI, non-Immunocheckpoint inhibitor; WBRT, whole brain radiotherapy; SRS, stereotactic radiosurgery; mOS, median overall survival; HR, hazard ratio; CI, confidence interval; NR not reported; lci, low confidence interval; uci, upper confidence interval; NSCLC, nonsmall-cell lung cancer.

### 3.2 Meta-analysis

**Figure 3:**
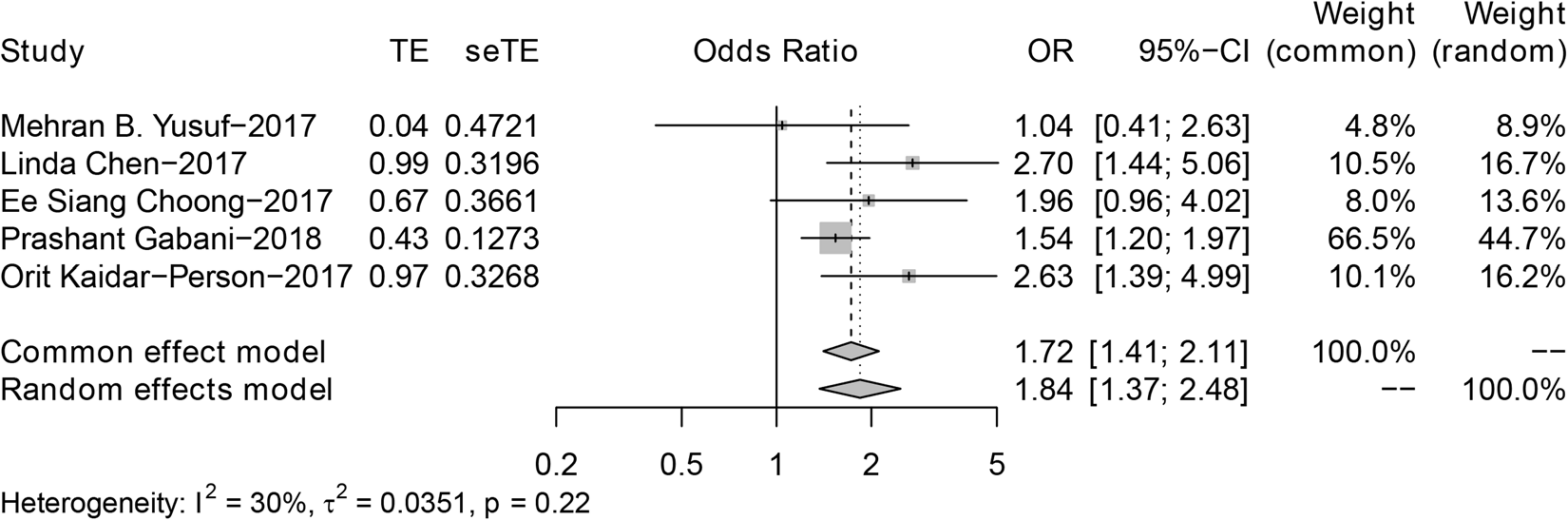
Forest plot of SRS + ICI vs ICI in OS.

Figure 3 shows the forest plot of SRS + ICI vs ICI for the article with OS as the endpoint. The results of the comprehensive fixed-effect model showed that the OS time of the SRS + ICI group was longer than that of the ICI group (hazard ratio, 1.72; 95% CI: 1.41–2.11; *P* = 0.22; *I* ^2^ = 30%). The use of immunotherapy combined with SRS was more beneficial to the survival time of BM patients. The combination of immunotherapy and radiotherapy was more effective than immunotherapy alone (Figure 3).

**Figure 4:**
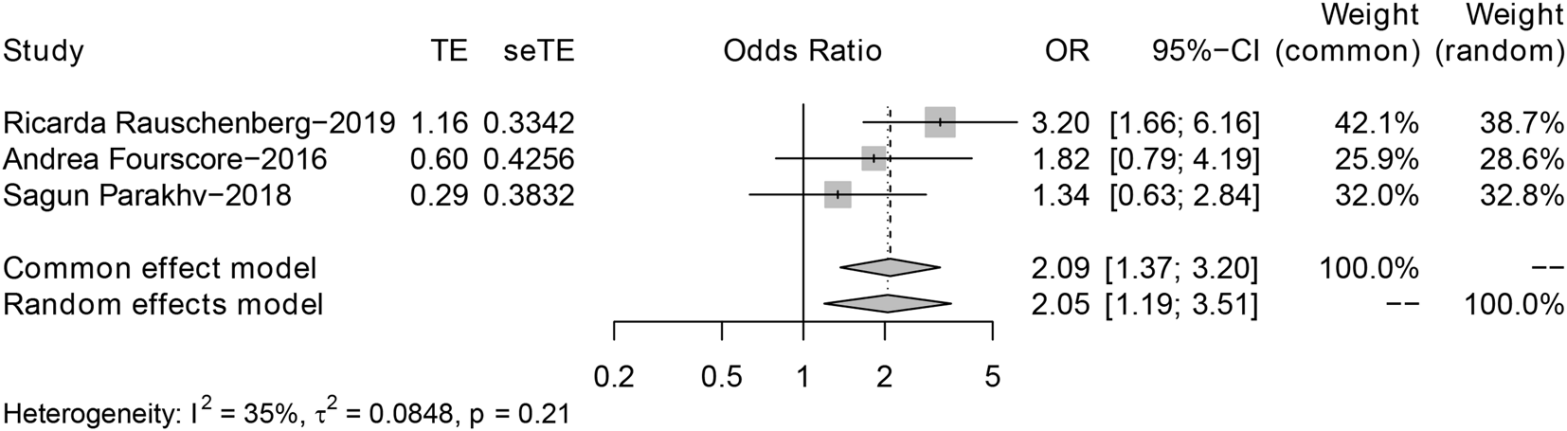
Forest plot of ICI vs Target in OS.

Figure 4 shows the forest map of ICI and Target with OS as the clinical endpoint. The combined fixed model showed that the survival time of ICI was longer than that of targeted therapy (hazard ratio, 2.09; 95% CI: 1.37–3.20; *P* = 0.21; *I* ^2^ = 35%). In particular, the use of immune checkpoints has significantly prolonged the clinical benefits of patients with advanced BM. It could be seen that in single drug comparison, immunotherapy had a more significant effect on survival prolongation than targeted therapy (Figure 4).

**Figure 5:**
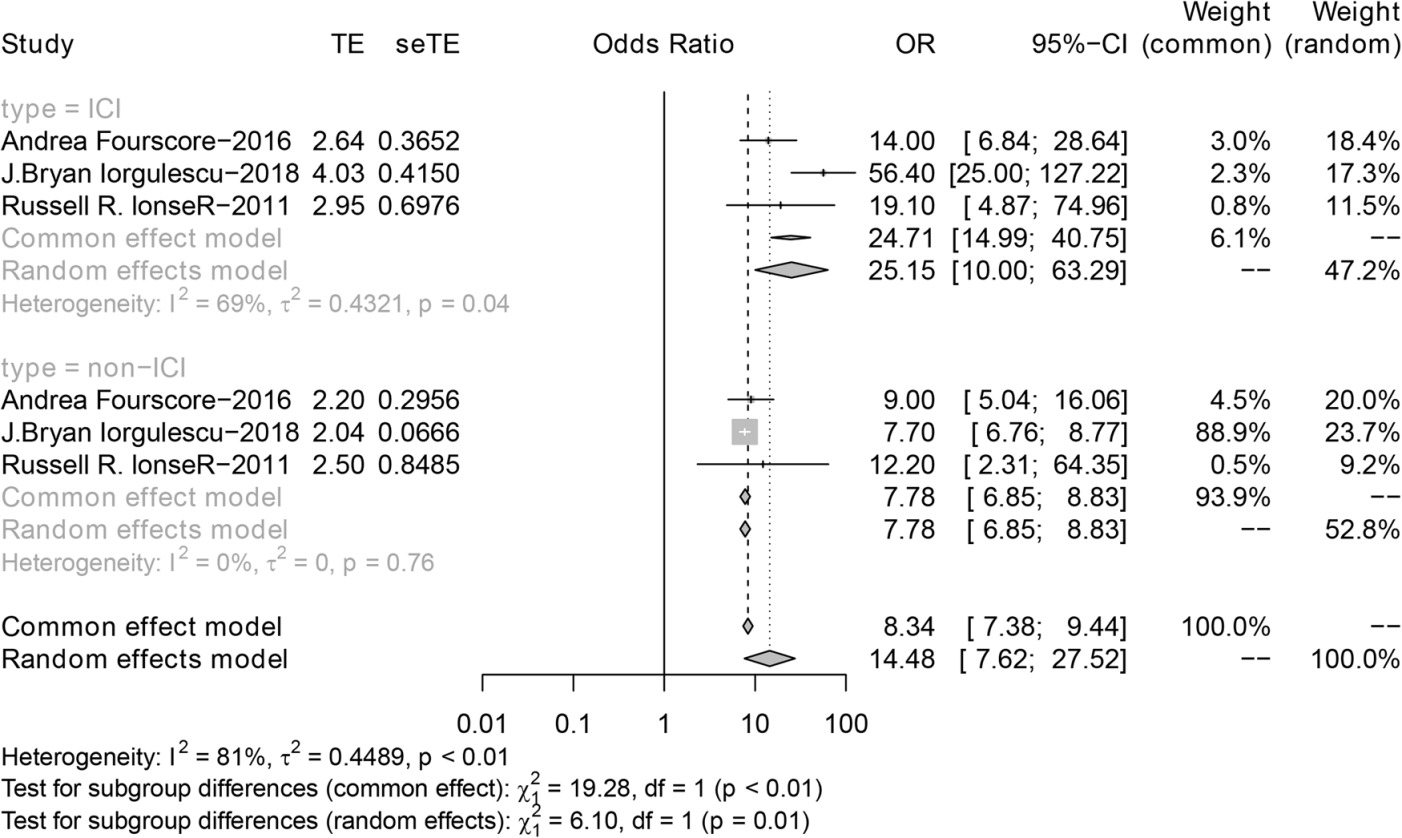
Forest plot of ICI vs non-ICI in OS.

The three articles included in Figure 5 compare specific survival lengths of ICI versus non-ICI. The summary survival time of the ICI group was 25.15 months, which was longer than that of the non-ICI group (hazard ratio, 7.78; 95% CI: 6.85–8.83). The great advantage of ICI was seen in this subgroup analysis, more than doubling the duration of the non-ICI approach (Figure 5).

### 3.3 Publication bias and sensitivity analysis

**Figure 6:**
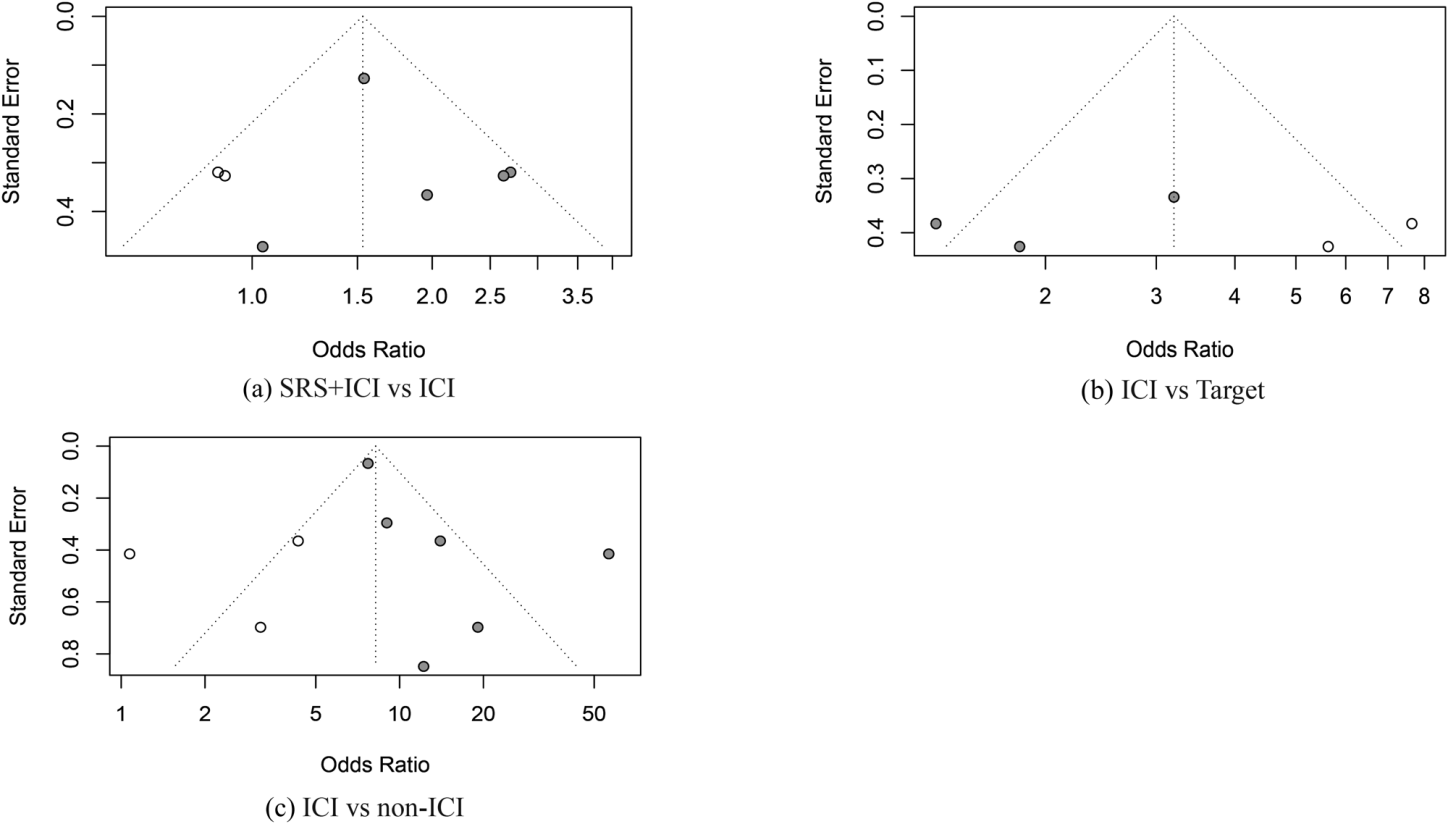
Funnel plot; (a) SRS + ICI vs ICI, (b) ICI vs Target, (c) ICI vs non-ICI.

**Figure 7:**
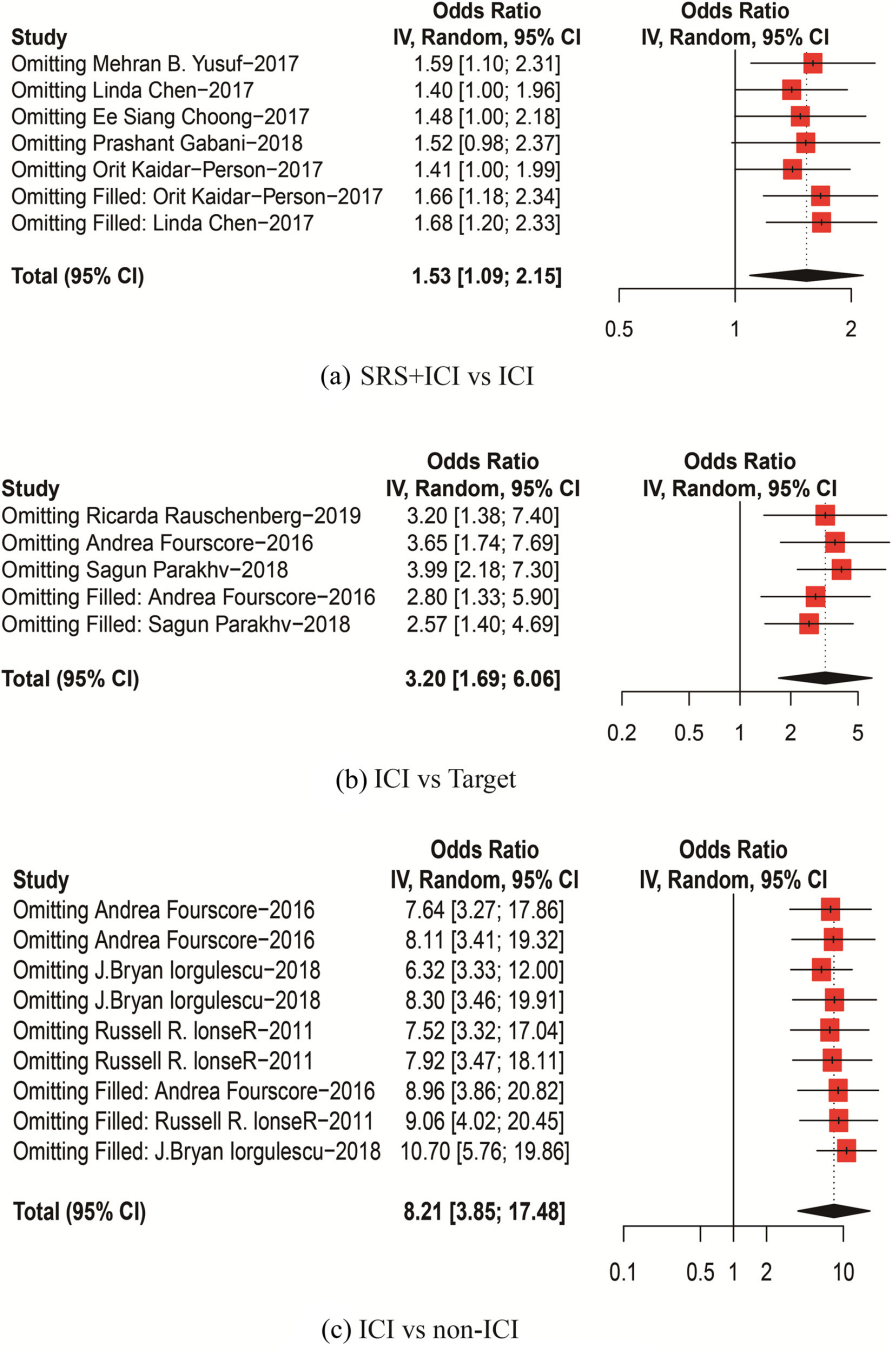
Sensitivity analysis; (a) SRS + ICI vs ICI, (b) ICI vs Target, (c) ICI vs non-ICI.

Our funnel plots and statistical tests showed no evidence of publication bias (Egger’s test; A.p =  0.479; B.p = 0.481; C.p = 0.107). Sensitivity analysis was run by excluding some high-risk articles, which did not significantly affect the results (Figures 6 and 7).

## 4 Discussion

The aim of this study was to investigate the differences in OS among BM patients treated with immunotherapy, radiotherapy, and molecularly targeted therapy. In cohort studies, patients receiving SRS combined with ICI had the longest survival in BM patients (hazard ratio, 1.72; 95% CI: 1.41 to 2.11; *P* = 0.22; *I* ^2^ = 30%). ICI also showed a great advantage versus non-ICI in immunotherapy of BM (hazard ratio, 25.15; 95% CI: 10.00–63.29 vs hazard ratio, 7.78; 95% CI: 6.85–8.83). Heterogeneity among studies was low, and the Egger test did not show obvious publication bias. Notably, most of the primary tumors of these BM were melanoma, followed by NSCLC and renal cell carcinoma. Since data on survival are scarce for tumors other than melanoma, subgroup analyses were not performed based on the primary tumor site.

Boire et al. suggested that the immune environment in the brain was different from the microenvironment of extracranial lesions [31]. The brain microenvironment was the core aspect that determines the development of BM. BM were also phenotypically adapted to the immune microenvironment. The BBB (blood–brain barrier) and blood–cerebrospinal fluid barrier were gatekeepers of the central nervous system that protect the brain from the potential consequences of inflammation [32]. Microglia can repair BBB damage during circulating tumor cell (CTC) migration, thus shielding the newly formed metastatic tumor [33]. Clinical experiments showed that cathepsin S (a protease that was usually expressed by leukocytes) mediated the migration of breast cancer cells through BBB [34]. Precisely because of this microenvironment, clinical trials were investigating targeted therapies that interrupt cancer–cell microenvironment interactions. Modulating the immune system through systemic administration of combined immune checkpoint blockade had an obvious clinical benefit for approximately half of the patients with melanoma that has metastasized to the brain [35]. Because half of the patients still did not benefit from ICI, further experiments were needed to explore the best way to regulate the immune system of BM patients. Niesel et al. used the immune regulation induced by radiotherapy in mice to increase the number of cytotoxic T cells and prevent the induction of lymphocyte-mediated immunosuppression. It was concluded that radioimmunotherapy can significantly improve tumor control and prolong the median survival time of those with brain metastasis of breast cancer [36].

A single-arm trial validated the efficacy of a combination of lapatinib and capecitabine as first-line therapy in untreated breast cancer with BM, although nearly half of the patients in the targeted therapy group experienced grade 3 or more adverse events [37]. The HER2-specific tyrosine kinase inhibitor tucatinib had a survival benefit in the treatment of BM when used in combination with trastuzumab and capecitabine [38]. The median duration of progression-free survival was 7.8 months in the study group and 5.6 months in the placebo group. Common adverse reactions included nausea and diarrhea. Diossy et al. showed by analyzing BM and their matched primary breast cancers that breast cancer BM tend to have high homologous recombination defect scores on the basis of genomic aberrations compared with primary tumors [39]. It suggested that BM may be more sensitive to PARP inhibitors than their corresponding primary tumors. The PI3K-AKT-mTOR pathway may be a promising therapeutic option for patients with BM [40]. Results from multicenter clinical trials supported the antitumor effect of the combination of everolimus, lapatinib, and capecitabine in BM patients [41]

Prior to the large-scale use of ICI, BM management primarily involved neurosurgeons and radiation oncologists, and classical treatment strategies included whole brain radiation therapy (WBRT), surgery, and SRS alone or in combination [42]. SRS was an effective treatment for BM and did not cause neurocognitive deficits associated with whole-brain radiotherapy. A recent meta-analysis by Chen et al. showed that patients receiving SRS treatment for brain stem metastases (BSM) rarely died of BSM progression and often experienced symptomatic improvement [43]. The combination of SRS and ICI resulted in significant improvements in both the median and maximum measures of lesion response, shortening the time to initial response and prolonging the time to intracranial recurrence [44]. Borius et al. compared toxicity in contemporaneous groups with non-contemporaneous groups or SRS alone. They found no skin toxicity, no significant increase in bleeding rates, or radiation necrosis with significant clinical effects. They concluded that SRS combined with systemic therapy appeared to be safe [45]. Petrelli et al. reported that in BM patients from solid tumors, the addition of concurrent immunotherapy to brain RT improves survival and provides long-term control [46]. A total of 128 patients were included in Anita Mahajan’s study. The local tumor recurrence-free rate was 43% in the observation group at 12 months, and 72% in the SRS group (HR, 0.46, 95% Cl: 0.24–0.88; *p* = 0.015) [47]. Whole brain radiotherapy (WBRT) could lead to toxicity, which reduced the recurrence in the central nervous system, but it had not yet been shown to provide survival benefits [48]. In ASTRO guidelines, it was strongly recommended to use SRS to improve local control for patients with BM [49].

### 4.1 Strengths and limitations

The advantages of this study were that these findings were supported by the large sample size and the number of studies, and the heterogeneities between studies were low to medium. However, this study had several limitations. First, as a meta-analysis, patient-level confounders and competitive risks cannot be fully identified or explained. Second, only the clinical endpoint of the total survival period was included, and other indicators, such as progression-free survival and time-to-progression, were not included. Third, treatment-related toxic events were not included in the analysis. Fourth, most of the primary tumors included in the article were melanoma. The number of studies on other tumors was small, making it impossible to conduct subgroup analysis based on tumor species. Therefore, the level of evidence we propose was moderate.

## 5 Conclusions

In BM, immunotherapy alone had a higher survival benefit than targeted therapy alone (hazard ratio, 2.09; 95% CI: 1.37–3.20). The OS time of SRS combined with ICI was higher than that of immunotherapy alone (hazard ratio, 1.72; 95% CI: 1.41–2.11). Our analysis confirmed that immunotherapy alone showed a higher OS benefit in BM patients than targeted therapy alone. The total survival time of patients with SRS combined with ICI was higher than that of patients with single ICI.
